# Effects of different types of physical activities on self-esteem in typically developing children and adolescents: a systematic review and network meta-analysis

**DOI:** 10.3389/fpubh.2026.1860396

**Published:** 2026-08-13

**Authors:** Wenqian Li, Ying Zhu, Yu Yin, Zaiwen Wang, Chengjun Li, Qian Qin, Shuang Li, Yue Liu, Hongyu Wang

**Affiliations:** 1 School of Physical Education and Health, Guangxi Normal University, Guilin, China; 2 School of Design, Guangxi Normal University, Guilin, China; 3 Guangxi Science and Technology Normal University, Guangxi, China

**Keywords:** adolescents, children, physical activity, self-esteem, typically developing

## Abstract

**Background:**

Amid the escalating global mental health crisis among children and adolescents, low self-esteem has been recognized as a pivotal risk factor for the onset of psychological difficulties. Developing interventions that are both cost-effective and widely accessible to enhance self-esteem carries substantial public health significance. The present study aims to systematically evaluate the effects of various forms of physical activity on self-esteem in typically developing children and adolescents, and to establish a comparative ranking of their effectiveness.

**Methods:**

Following the PRISMA 2020 guidelines, randomized controlled trials (RCTs) were systematically retrieved from PubMed, the Cochrane Library, Embase, and Web of Science from database inception to March 31, 2026. The Cochrane Risk of Bias 2 (RoB2) tool was used to assess the risk of bias. A network meta-analysis was conducted to integrate both direct and indirect evidence, with standardized mean differences (SMD) and corresponding 95% confidence intervals (CI) calculated. The surface under the cumulative ranking curve (SUCRA) was applied to rank the relative effectiveness of interventions.

**Results:**

A total of 17 RCTs were ultimately included. The network meta-analysis indicated that resistance exercise showed the highest probability of being the most effective intervention for improving self-esteem (SUCRA = 97.8%), and was significantly superior to multimodal exercise, stretching exercise, and no intervention; while its advantages over aerobic exercise and mind–body exercise did not reach statistical significance. Funnel plot revealed no significant asymmetry (low publication bias). Combined with good consistency and low study bias, our findings are robust.

**Conclusion:**

Resistance exercise, aerobic exercise, mind–body exercise, multimodal exercise, and stretching exercise were all effective in enhancing self-esteem among typically developing children and adolescents, with resistance exercise showing the highest probability of being the most effective. These findings suggest that resistance exercise may serve as the preferred physical activity intervention to promote self-esteem in school, family, and community settings based on current evidence.

**Systematic review registration:**

https://www.crd.york.ac.uk/prospero/display_record.php?ID=CRD420261364418, PROSPERO (CRD420261364418).

## 1 Introduction

With the increasing global attention to the physical and mental health of children and adolescents, psychological problems in this population have emerged as a prominent global public health challenge, with profound societal implications (1–3). Low self-esteem has been identified as a critical precursor and risk factor that can trigger and exacerbate various internalizing and externalizing psychological problems, as well as increase the risk of developing mental disorders (4). This global mental health crisis among children and adolescents extends far beyond individual and family well-being, becoming a key issue that impacts national education policy implementation, public health system development, and the sustainable development of human capital. It also underscores the urgent need to explore low-cost and widely accessible interventions aimed at enhancing self-esteem (5, 6).

Self-esteem, defined as an individual’s subjective evaluation and acceptance of their own value, abilities, and self-identity, is a multidimensional construct encompassing global self-esteem, physical self-concept, and social self-concept (7).

It constitutes a fundamental cornerstone of psychological development in children and adolescents and serves as a critical protective factor shaping their lifelong mental health trajectories (7). Notably, different validated assessment instruments may measure distinct facets of self-esteem, with some focusing on global self-evaluation and others targeting domain-specific dimensions such as physical competence or social acceptance (8). Healthy levels of self-esteem not only support children and adolescents in establishing a stable sense of self, adapting to school and social environments, and maintaining healthy interpersonal relationships, but also buffer against the psychological impact of adversity and stress encountered during development (9). Conversely, persistent low self-esteem during childhood and adolescence can initiate a self-reinforcing vicious cycle: negative self-perception leads to maladaptive coping behaviors, which further worsen mental health outcomes and reinforce negative self-views (10). Therefore, promoting healthy self-esteem development among typically developing children and adolescents is not only a key entry point for the early prevention of psychological problems but also a central scientific concern for fostering holistic development in this population and alleviating the long-term burden on families and public health systems.

Current interventions to enhance self-esteem in children and adolescents primarily include psychological, educational, and family-based approaches (11). While these interventions have demonstrated efficacy for clinical populations, they face significant scalability challenges: psychological interventions rely heavily on specialized professionals and are costly to implement widely (12); educational interventions are limited by variable teacher training and program standardization (13); and family-based interventions depend heavily on parental capacity and engagement, leading to inconsistent outcomes (14).

Physical activity, defined by the World Health Organization (WHO) as any skeletal muscle movement that expends energy, is a broad concept that technically includes low-intensity daily activities such as writing. However (15), consistent with the intervention inclusion criteria in this study, we specifically focus on purposeful, structured physical activities with measurable intensity, frequency, and duration, including aerobic exercise, resistance exercise, mind–body exercise, multimodal exercise, and stretching exercise, which are the core interventions evaluated in this meta-analysis (15). Physical activity promotes self-esteem in children and adolescents through a multidimensional, synergistic mechanism (16). At the physiological level, regular physical activity can improve physical fitness and body composition, enhance body satisfaction, and regulate neurotransmitter and stress hormone secretion, thereby alleviating negative emotions and laying a physiological and emotional foundation for self-esteem development (17). At the psychological level, the sense of control and goal achievement experienced during exercise can directly enhance self-efficacy, correct negative self-cognitions, and low-stress failure scenarios foster resilience, further strengthening stable self-evaluation (18). At the social level, collective physical activities such as team sports create abundant opportunities for peer interactions, from which individuals can derive a sense of belonging, positive social feedback, and social recognition, thus reinforcing their sense of self-worth (19). It is noteworthy that different types of physical activity exhibit significant differences in terms of exercise modality, social attributes, and intensity characteristics. Consequently, the intervention effects on self-esteem through the aforementioned three pathways may also vary markedly (20).

Previous meta-analyses have confirmed that physical activity positively influences self-esteem in children and adolescents (21, 22). However, key evidence gaps remain. First, most studies have only compared physical activity with no intervention, lacking systematic comparisons and hierarchical evaluation of different activity types, which limits identification of the optimal intervention for enhancing self-esteem in typically developing youth (21). Second, existing research often focuses on clinical populations, such as children with developmental disorders, obesity, or psychiatric conditions, resulting in insufficient high-quality evidence for typically developing children and adolescents and restricting the generalizability of findings (22).

Notably, focusing exclusively on typically developing children and adolescents carries critical theoretical and public health significance (2). First, baseline self-esteem differs fundamentally between groups: clinical populations have significantly lower baseline levels with greater room for improvement, whereas typically developing youth exhibit normative, stable self-esteem where a “ceiling effect” makes intervention effects more difficult to detect, allowing for a more accurate assessment of the intrinsic efficacy of different physical activity types (16). Second, the mechanisms of self-esteem enhancement diverge: improvements in clinical groups often accompany symptom alleviation, while in typically developing youth, they stem from normative developmental processes such as competence acquisition and social integration, which cannot be fully inferred from clinical studies (11). Third, as the vast majority of the global youth population, typically developing children and adolescents form the first line of defense against the mental health crisis (4). Evidence specific to this group is essential for developing universal, scalable prevention strategies for schools and communities.

To address these gaps, the present study targets typically developing children and adolescents and employs a systematic review with network meta-analysis. By including rigorously selected high-quality RCTs, it evaluates the effects of different types of physical activity on self-esteem and establishes a hierarchical ranking of their relative efficacy. This study aims to answer the core scientific question: What are the relative effects and rankings of different physical activity types in improving self-esteem among typically developing children and adolescents?

## 2 Methods

### 2.1 Protocol and registration

This systematic review was conducted in strict accordance with the Preferred Reporting Items for Systematic Reviews and Meta-Analyses (PRISMA) 2020 Statement (73), and fully adhered to all requirements of the statement regarding study inclusion criteria, data organization procedures, statistical analysis methods, and result reporting standards. The study protocol was prospectively registered with the International PROSPERO, with the unique identification number CRD420261364418.

### 2.2 Data sources and search strategy

This study conducted a systematic and comprehensive search of four major electronic databases: PubMed, Cochrane Library, Embase, and Web of Science, to identify studies examining the effects of different types of physical activity on self-esteem in typically developing children and adolescents. To ensure the completeness of the literature search, reference lists of included studies were manually searched for potentially eligible studies. The search covered the period from the inception of each database through March 31, 2026. The search strategy was developed based on the PICOS principles (23): the target population consisted of typically developing children and adolescents; the intervention was various types of physical activity; the control group included any type of comparison group; the outcome measure was self-esteem-related assessments; and only RCTs were included. The primary search terms included “adolescents” or “children” or “physical activity” or “aerobic exercise” or “self-esteem” or “self-worth” or “RCT”.

### 2.3 Study selection

After implementing the above search strategy, two researchers (HYW and YZ) independently conducted the literature screening following the PRISMA guidelines.

Inter-rater reliability was assessed using Cohen’s kappa coefficient at both the title/abstract screening stage and full-text screening stage. Agreement was classified as moderate (0.40–0.59), good (0.60–0.74), and excellent (>0.75) (24). In the initial screening phase, titles and abstracts were reviewed, and studies potentially meeting the predefined inclusion criteria were selected. For studies that met the preliminary screening criteria, full-text retrieval and critical evaluation were carried out. The studies ultimately included in the quantitative synthesis were confirmed through team discussions and reached a consensus. Throughout this process, disagreements were resolved through multiple rounds of iterative communication until a unified conclusion was reached.

### 2.4 Inclusion and exclusion criteria

The study selection criteria for this systematic review were developed based on the PICOS framework.

Studies meeting all of the following criteria were included:

1. Study design: RCTs.
2. Participants: Typically developing children and adolescents, defined as individuals without diagnosed physical illnesses (e.g., chronic diseases, physical disabilities) or clinical psychological disorders (e.g., major depression, anxiety disorders, developmental disorders).

Interventions: Purposeful, structured physical activities consistent with the definition in the Introduction, including aerobic exercise, mind–body exercise, resistance exercise, multimodal exercise, and stretching exercise, with standardized descriptions of intensity (e.g., %HR<sub>max</sub>, Metabolic Equivalents [METs]), frequency (sessions/week), and duration.

1. Outcomes: Data measuring self-esteem before and after the intervention using validated scales.
2. Data completeness: Sufficient original or extractable data (including means, standard deviations, sample sizes, and precise *p*-values) to calculate effect sizes (e.g., SMD).
3. Language: Only English-language articles were included to ensure study quality and avoid translation bias.

Studies were excluded if they met any of the following criteria:

1. Study design: Observational studies (e.g., cross-sectional studies, case–control studies, cohort studies).
2. Participants: Were limited to overweight/obese children and those with confirmed physical or psychological clinical diagnoses. Subclinical psychological characteristics (e.g., low self-concept) or social environmental factors (e.g., left-behind status) were not considered exclusion criteria, as these do not constitute clinical disorders and are prevalent in the general pediatric population.
3. Interventions: Non-physical activity interventions, interventions with insufficient details (e.g., unquantifiable intensity/duration), or combined interventions whose effects are difficult to distinguish.
4. Study type: Qualitative studies, reviews, or conference proceedings.

Data completeness: Lack of critical outcome data or data that could not be extracted (e.g., descriptive statistics without numerical values).

1. Ethical issues: Violations of ethical standards (e.g., lack of informed consent, disproportionate risk–benefit ratio).
2. Language: Publications in non-English languages, unpublished preprints, or standalone abstracts without full-text peer-reviewed versions.

### 2.5 Quality assessment

We employed the Cochrane Risk of Bias 2 (RoB2) assessment tool to evaluate study quality across five domains (25): (1) randomization process; (2) deviations from intended interventions; (3) missing outcome data; (4) measurement of outcomes, and (5)selection of reported results. Based on these criteria, each study was systematically evaluated for overall bias and classified into one of three categories: low risk, high risk, or some concerns. We employed the RoB2 assessment tool to evaluate study quality across five domains: (1) randomization process; (2) deviations from intended interventions; (3) missing outcome data; (4) measurement of outcomes, and (5) selection of reported results. Based on these criteria, each study was systematically evaluated for overall bias and classified into low risk, some concerns, or high risk. Specifically, adequate randomization and allocation concealment were rated low risk, unclear randomization was rated some concerns, and non-random grouping or severe baseline imbalance was rated high risk. Strict protocol implementation with balanced adherence was low risk for intervention deviations; unavoidable unblinding without systematic bias was some concerns, and arbitrary protocol changes or unbalanced group crossover were high risk. Balanced low attrition and complete outcome data were low risk, unrelated moderate missing data was some concerns, and outcome-relevant unbalanced high attrition was high risk.

### 2.6 Statistical analysis

For continuous self-esteem outcomes, standardized mean differences (SMD) with 95% confidence intervals (CI) were calculated to pool effect sizes across studies using different measurement scales, where a positive SMD indicates greater improvement in self-esteem in the intervention group. Statistical heterogeneity was evaluated using the chi-square test and I^2^ statistic, with I^2^ > 50% defined as moderate heterogeneity and I^2^ > 75% defined as high heterogeneity. An inverse variance-weighted random-effects model incorporating τ^2^ estimation was used to pool effect sizes, as it explicitly accounts for between-study heterogeneity arising from variations in intervention protocols and assessment tools, providing more conservative and generalizable results than a fixed-effects model. Consistent with PRISMA-NMA guidelines, a frequentist framework was adopted rather than a Bayesian approach to enhance result interpretability and avoid computational convergence issues associated with Markov chain Monte Carlo methods. Network meta-analysis was performed using the “network” package in Stata 15.1, following three core steps: (1) Construction of an evidence network plot, where node size corresponds to total sample size and line thickness represents the number of direct comparisons between interventions; (2) Pooling of effect sizes using maximum-likelihood multivariate meta-regression to integrate both direct and indirect evidence; (3) Node-splitting test to assess consistency between direct and indirect effect estimates for each pairwise comparison, with *p* > 0.05 indicating no statistically significant inconsistency. Notably, the evidence network of this study presents a star-shaped structure with partial pairwise direct comparisons. All five physical activity modalities have direct comparisons with the no-intervention control, while resistance exercise, mind–body exercise, and multimodal exercise also have direct comparisons with stretching exercise. For intervention pairs without direct head-to-head RCTs (e.g., aerobic exercise vs. resistance exercise), their effect sizes were calculated via indirect comparisons using the shared common control (no intervention), which is a standard application scenario of network meta-analysis. The surface under the cumulative ranking curve (SUCRA) was calculated to rank interventions by relative efficacy. SUCRA values represent the probability that an intervention ranks better than all other comparators, derived from the cumulative sum of probabilities of an intervention being ranked at each possible position, standardized to a 0–100% scale. A higher SUCRA value indicates greater relative efficacy: 100% means an intervention is consistently ranked first, while 0% means it is consistently ranked last.

## 3 Results

### 3.1 Trial selection

**Figure 1:**
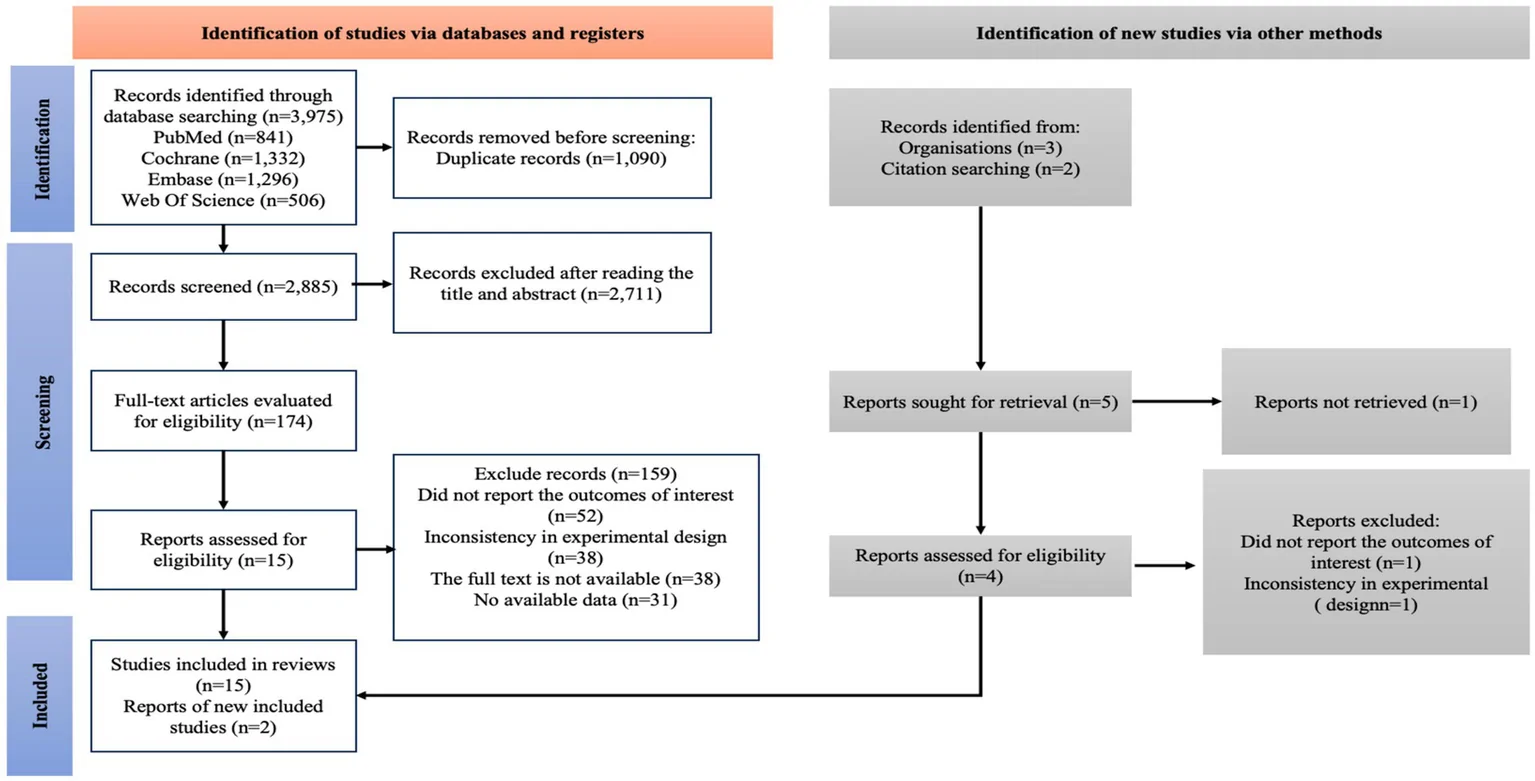
A summary of the evidence searches and selection process.

To ensure the reliability of the literature search and screening process, two researchers (ZWW and WQL) independently reviewed the titles, abstracts, and full texts of the retrieved articles. Cohen’s kappa coefficient was calculated to assess inter-rater reliability at two screening stages. In the initial search, a comprehensive search was conducted across four electronic databases up to March 31, 2026, yielding a total of 3,975 articles. After removing duplicates (*n* = 1,090), 2,885 articles remained for further evaluation. During the title and abstract screening, 2,711 articles were excluded, leaving 174 articles for full-text review. At this stage, inter-rater reliability between the two assessors was considered “good” (Cohen’s kappa = 0.71). Following full-text review, an additional 159 articles were excluded for the following reasons: 52 did not report the outcomes of interest, 38 had inconsistent study designs, 38 had no available full-text versions, and 31 had insufficient usable data. Only 15 articles met the predefined inclusion criteria. An additional 2 eligible studies were further identified through manual screening of the reference lists of these articles. Thus, a total of 17 studies were included in the final analysis (Figure 1). At this stage, inter-rater reliability between the two assessors was rated as “excellent” (Cohen’s kappa = 0.79).

### 3.2 Trial characteristics

Table 1 summarizes the characteristics of the studies included in this analysis. These studies were published between 2005 and 2026 (26–42). Sample sizes in the intervention groups ranged from 21 to 7,860 participants, while those in the control groups ranged from 15 to 8,157 participants. The included studies covered typically developing children and adolescents aged 6–19 years. With an overall weighted mean age of approximately 12.3 years across all participants.

**Table 1:** Summary table of included reviews.

Study	Country	N (IG; CG)	Age (IG; CG)	Intervention (IG)			Intervention (CG)			Population	Outcomes
				Intervention content	Intervention time, frequency, period	Type	Intervention content	Intervention time, frequency, period	Type		
Avan (2026) (26)	Türkiye	42; 42	19.14 ± 0.91; 19.14 ± 0.91	Physical Activity Health Education, and Progressive Exercise	20–60 min, 2 weekly, 12 weeks	Aerobic exercise	No intervention	12 weeks	No intervention	Late adolescents	PWBS
Gourlan et al. (2025) (31)	France	1,420; 1,303	9.1 ± 0.9; 9.1 ± 0.9	7 types of mixed exercises	100–120 min, 6 week each year, 16 mouths	Multimodal exercise	Maintain daily life	16 mouths	No intervention	Children	SES
Cowley et al. (2021) (32)	United Kingdom	22; 20	14.2 ± 1.1; 14.2 ± 1.1	Live online group workouts and Self-designed workouts	30 min, 3 weekly, 6 weeks	Multimodal exercise	Usual care	6 weeks	No intervention	Adolescent girls	SCS
Li et al. (2025) (40)	China	30; 30	10.73 ± 1.99; 11.20 ± 1.24	Cha-cha Latin dance training	45 min, 5 weekly, 12 weeks	Stretching exercises	Usual care	12 weeks	No intervention	Children with low self-concept	PHCSCS
Telles et al. (2013) (36)	India	49; 49	8–13; 8–13	Yoga	45 min, 5 weekly, 3 mouths	Mind–body exercise	Physical exercise	45 min, 5 weekly, 3 mouths	No intervention	Healthy children	SEIC
Li and Zhou (2025) (27)	China	30; 30	14–18; 14–18	High-Intensity Interval Training(e.g., 30 s high intensity + 30 s rest)	15 min, 5 weekly, 8 weeks	Aerobic exercise	Maintain daily life	15 min, 5 weekly, 8 weeks	No intervention	Adolescents	MSAPS
Scafuto et al. (2025) (33)	Italy	168; 118	10–19; 10–19	Gaia(Including body self-awareness and self-confidence exercises)	90 min, 1 weekly, 12 weeks	Multimodal exercise	No intervention	12 weeks	No intervention	Adolescents	PWBS
Burgess et al. (2006) (34)	United Kingdom	25; 25	13–14; 13–14	Aerobic dance first, followed by physical education class (swimming)	50 min, 2 weekly, 6 weeks	Multimodal exercise	Physical education class (swimming) first, followed by aerobic dance	50 min, 2 weekly, 6 weeks	Stretching exercises	Schoolgirls	CY-PSPP
Smith et al. (2018) (42)	Australia	353; 254	14.1 ± 0.4; 14.2 ± 0.5	Resistance exercise	90 min, 1 weekly, 10 weeks	Resistance exercise	Usual care	10 weeks	No intervention	Adolescents	PSDQ
Eather et al. (2016) (41)	Australia	51; 45	15.4 ± 0.5; 15.4 ± 0.5	The CrossFit ™ Teens Resistance exercise program	60 min, 2 dayly, 8 weeks	Resistance exercise	Sport lesson+HPE lesson	60 min, 2 dayly, 8 weeks	Stretching exercises	Adolescents	PSDQ
Annesi (2005) (35)	United States	50; 42	9–12; 9–12	Resistance and aerobic exercise program	45 min, 3 weekly, 12 weeks	Multimodal exercise	No exercise	12 weeks	No intervention	Healthy children	SCS
Wassenaar et al. (2021) (28)	United Kingdom	7,860; 8,157	12.5(0.296); 12.5(0.293)	High-intensity interval training-style vigorous physical activity	60 min, 2 weekly, 10 mouths	Aerobic exercise	Maintain daily life	2 weekly, 10 mouths	No intervention	Young adolescents	PSDQ
Noggle et al. (2012) (37)	United States	36; 15	17.1(0.6); 17.3(0.8)	Kripalu-based yoga program	30–40 min, 2–3 weekly, 10 weeks	Mind–body exercise	Maintain daily life	30–40 min, 2–3 weekly, 10 weeks	No intervention	High school students	IPPA
Halliwell et al. (2018) (38)	United Kingdom	190; 154	9.34; 9.34	Yoga	40 min, 1 weekly, 4 weeks	Mind–body exercise	Maintain daily life	2 weekly, 4 weeks	No intervention	Healthy children	SCS
Bao and Jin (2015) (39)	China	73; 69	14.79(0.69); 14.65(0.64)	Tai Chi	60 min, 5 weekly, 1 year	Mind–body exercise	Broadcast Gymnastics	60 min, 5 weekly, 1 year	Stretching exercises	Year 7 pupils	SCS
Costigan et al. (2017) (29)	Australia	21; 22	15.7 (0.7); 15.6 (0.6)	Aerobic exercise program	8–10 min, 3 weekly, 8 weeks	Aerobic exercise	Usual care	8 weeks	No intervention	Adolescents	PSDQ
Costigan et al. (2017) (29)	Australia	22; 22	15.5 (0.6); 15.6 (0.6)	Resistance and aerobic exercise program	8–10 min, 3 weekly, 8 weeks	Resistance exercise	Usual care	8 weeks	No intervention	Adolescents	PSDQ
Fernández-Sánchez et al. (2025) (30)	Spain	276; 286	9–11; 9–11	Traditional playground game-based HIIT	60 min, 4 weekly, 8 mouths	Aerobic exercise	Usual care	50 min, 2 weekly, 8 mouths	No intervention	Healthy children	KIDSCREEN-27

IG: intervention group; CG: control group; N: Number; PWBS: Psychological Well-Being Scale; SES: Rosenberg Self-Esteem Scale; SCS: Self-Concept Scores; PHCSCS: Piers-Harris Children’s Self-Concept Scale; SEIC: Indian adaptation of Battle’s Self-Esteem Inventory for Children; MSAPS: Multidimensional Scale of Adolescent Psychological State; CY-PSPP: Children and Youth Physical Self-Perception Profile; PSDQ: Physical Self-Description Questionnaire; IPPA: Inventory of Positive Psychological Attitudes; KIDSCREEN-27: The KIDSCREEN-27 Health-Related Quality of Life Questionnaire.

To examine whether different types of physical activity exert differential effects on self-esteem in typically developing children and adolescents, we categorized all included interventions into five mutually exclusive types based on established classification frameworks from previous high-quality network meta-analyses (43, 44):

Aerobic exercise (5 studies): Characterized by moderate-to-low intensity, continuous rhythmic movements, with the primary objective of improving cardiorespiratory fitness and overall metabolic function (26–30). Multimodal exercise (5 studies): Integrated interventions combining two or more distinct movement forms, emphasizing coordination, participant engagement, and training continuity, with a moderate exercise load well-tolerated by children and adolescents (31–35). Mind–body exercise (4 studies): Focused on the coordinated integration of bodily movements, diaphragmatic breathing, and intentional mental focus, emphasizing the balance between activity and stillness and the unity of body and mind, with gentle and fluid movement patterns (36–39). Stretching exercise (4 studies): Centered on slow, controlled elongation of muscles, tendons, and joints, with the core purpose of improving body flexibility and local blood circulation (34, 39–41). Resistance exercise (3 studies): Performed against body weight or external loads, with the primary goal of enhancing muscular strength and bone density, featuring diverse movement forms and high participant engagement (29, 41, 42).

Notably, all included RCTs only adopted no-intervention or usual care as control conditions; no other non-physical activity interventions (e.g., psychological counseling, educational programs, pharmacological treatments) were included as comparators. This network meta-analysis enabled both direct and indirect pairwise comparisons among the five types of physical activity, as well as head-to-head comparisons between each physical activity modality and the no-intervention control group. Commonly used instruments to assess self-esteem in typically developing children and adolescents included the Psychological Well-Being Scale (PWBS), Rosenberg Self-Esteem Scale (SES), Self-Concept Score (SCS), Piers-Harris Children’s Self-Concept Scale (PHCSCS), Self-Esteem Inventory for Children (SEIC), Multidimensional Scale of Adolescent Psychological State (MSAPS), Psychological Well-Being Scale (PWB), Children and Youth Physical Self-Perception Profile (CY-PSPP), Physical Self-Description Questionnaire (PSDQ), and the Inventory of Positive Psychological Attitudes (IPPA).

### 3.3 Risk of bias

Among the 17 included studies, the risk of bias was assessed across five domains using the Cochrane Risk of Bias 2 (RoB2) tool, with the following results: Randomization process: 11 studies were rated as low risk (adequate random sequence generation and allocation concealment), 4 were rated as having some concerns (insufficient details on randomization procedures), and 2 were rated as high risk (non-random grouping or severe baseline imbalance). Deviations from intended interventions: 10 studies were rated as low risk (strict protocol implementation with balanced adherence), 5 were rated as having some concerns (unavoidable unblinding without systematic bias), and 2 were rated as high risk (arbitrary protocol changes or unbalanced group crossover). Missing outcome data: 12 studies were rated as low risk (balanced low attrition and complete outcome data), 4 were rated as having some concerns (unrelated moderate missing data), and 1 was rated as high risk (outcome-relevant unbalanced high attrition). Measurement of outcomes: All 17 studies were rated as low risk (validated self-esteem scales used with standardized assessment procedures). Selection of reported results: All 17 studies were rated as low risk (pre-specified outcomes fully reported without selective reporting).

**Figure 2:**
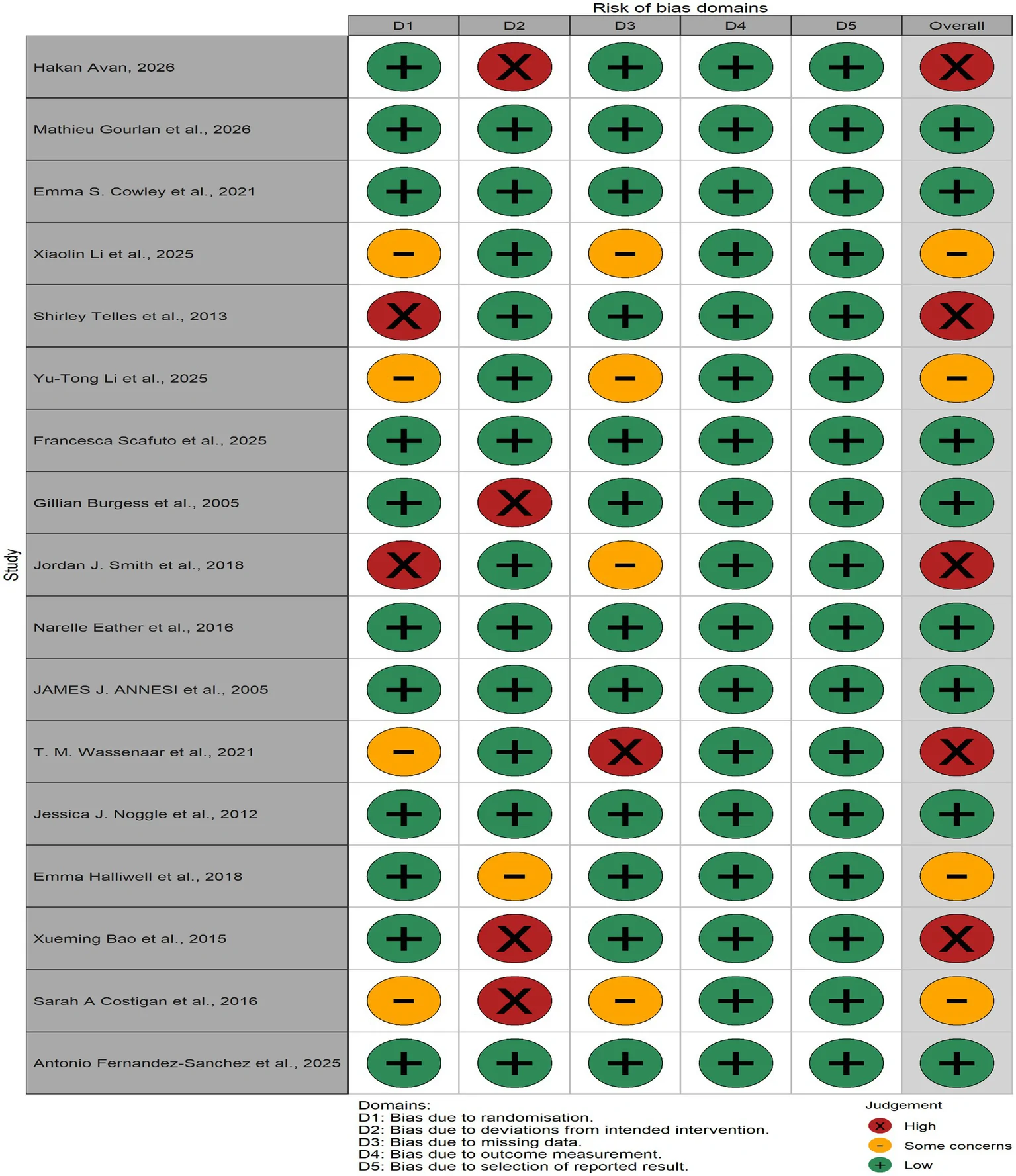
Risk of bias of included studies.

**Figure 3:**
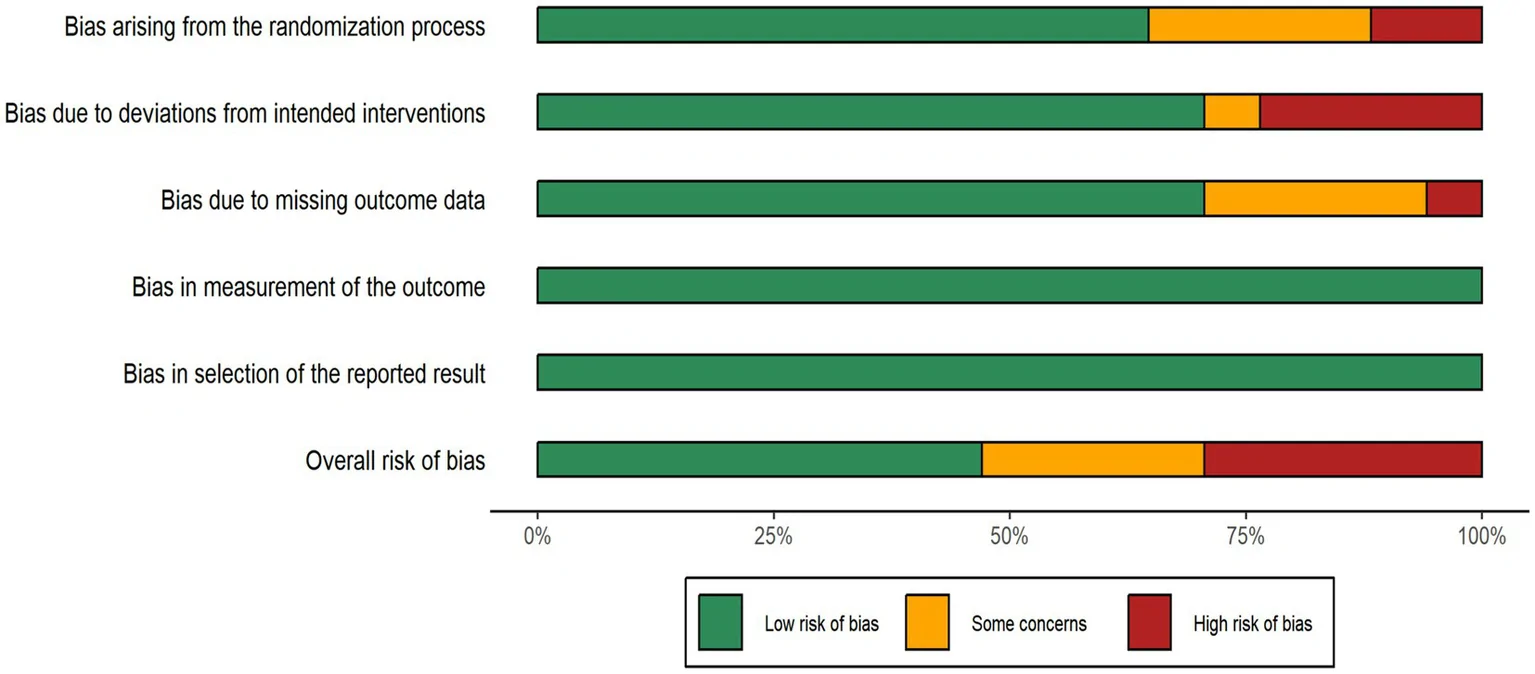
Risk of bias assessment of included studies.

Based on the overall risk of bias assessment, 8 studies were classified as low risk, 4 as having some concerns, and 5 as high risk. The detailed risk of bias assessment results for each study are presented in Figures 2, 3.

### 3.4 Network meta-analysis

**Figure 4:**
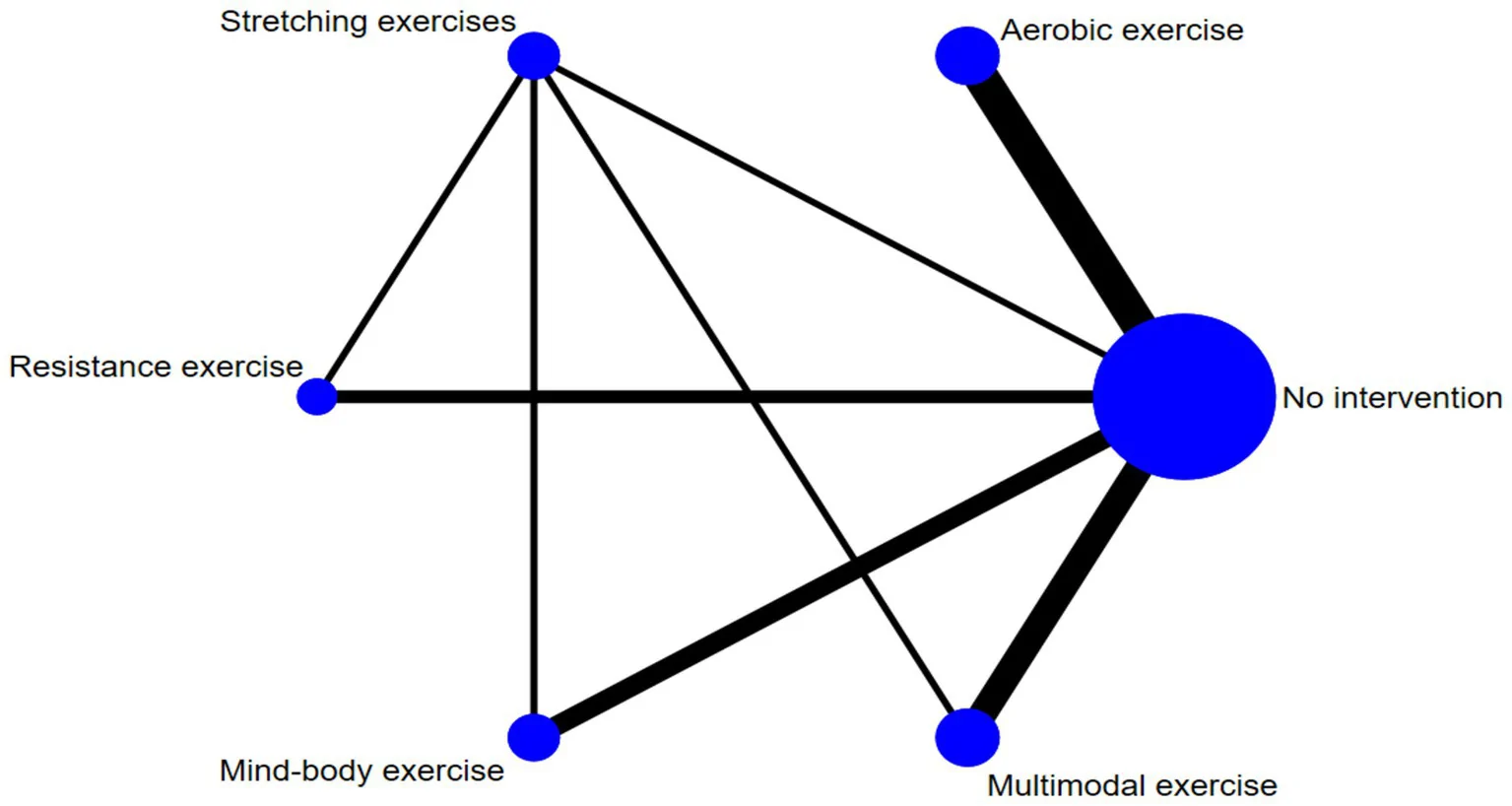
Network diagram of direct comparisons between interventions. Node size corresponds to the total sample size of each intervention, and line thickness represents the number of direct head-to-head RCTs between two interventions. Note that the absence of a line between two interventions does not mean no comparative evidence exists; indirect comparative evidence via shared common controls is integrated in the network meta-analysis.

Figure 4 presents the network meta-analysis diagram. The three interventions with the largest sample sizes in the intervention groups were aerobic exercise, multimodal exercise, and mind–body exercise. The most commonly studied comparisons included traditional aerobic exercise versus no intervention, and multimodal exercise versus no intervention. Heterogeneity assessment revealed a moderate overall between-study variance with τ^2^ = 0.89, and the pooled I^2^ statistic was 58.2%, indicating moderate statistical heterogeneity across included studies, which was primarily attributed to variations in intervention duration, frequency, intensity, and self-esteem assessment tools. The node-splitting test confirmed no significant inconsistency between direct and indirect evidence for all pairwise comparisons (all *p* > 0.05), supporting the validity of the network meta-analysis results.

**Figure 5:**
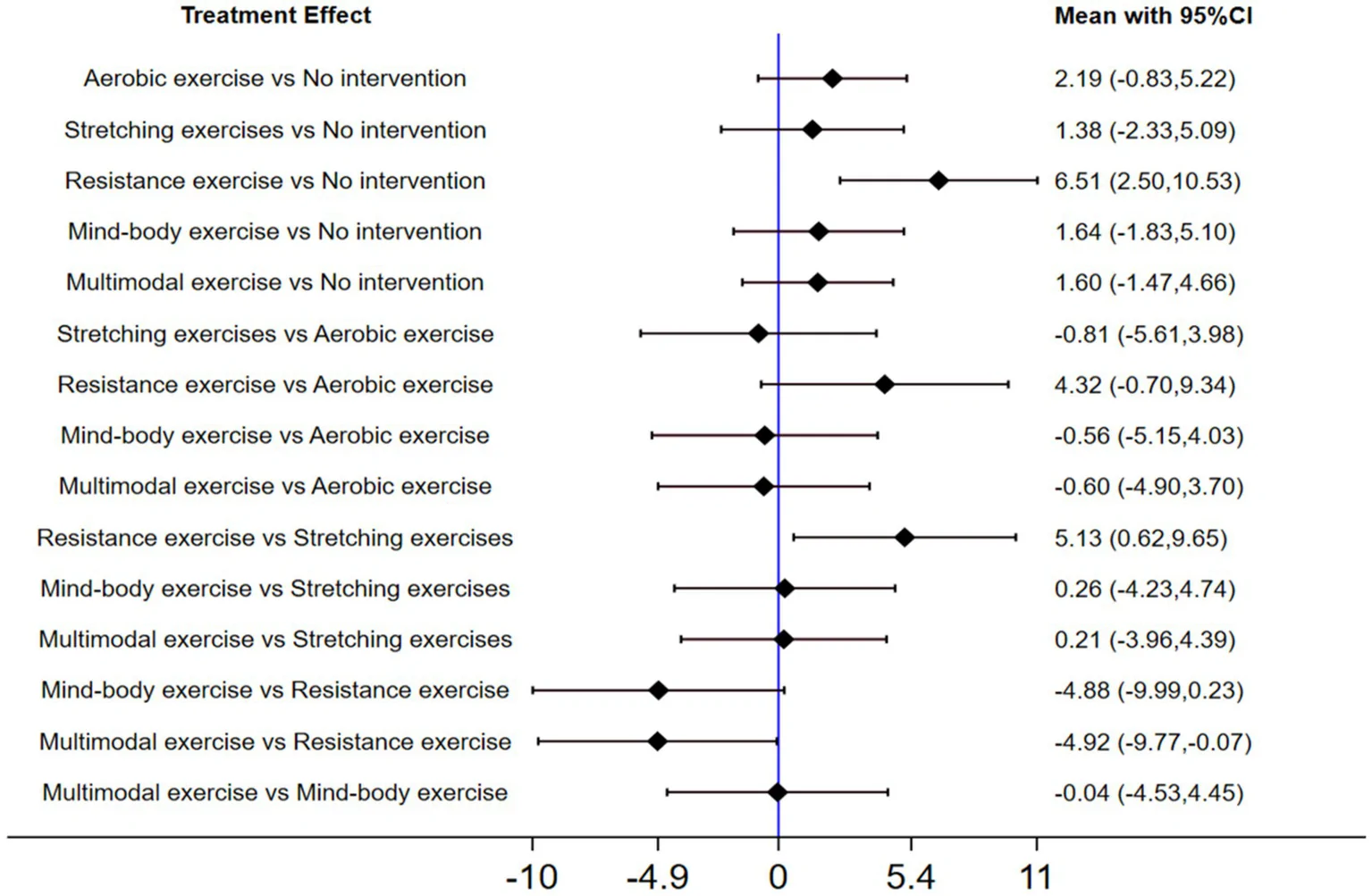
Network meta-analysis forest plot. Physical activity vs. no-intervention control; Pairwise comparisons among physical activity types. All effect sizes presented are pooled results integrating both direct and indirect evidence from the network meta-analysis.

Figure 5 presents network meta-analysis forest plots illustrating the effects of different physical activity interventions on self-esteem. Panel Figure 5a shows direct comparisons between each physical activity modality and the no-intervention control group. Panel Figure 5b displays pairwise comparisons among the five types of physical activity, integrating both direct and indirect evidence from the network meta-analysis.

SMD were calculated based on change scores from pre- to post-intervention (intervention group vs. control group); positive SMD values indicate greater advantage in self-esteem for the first-mentioned intervention. All pairwise comparison results are presented in detail in Table 2. Notably, resistance exercise showed significant improvements over multimodal exercise [SMD = 4.92 (95% CI: 0.07, 9.77)], stretching exercise [SMD = 5.13 (95% CI: 0.62, 9.65)], and no intervention [SMD = 6.51 (95% CI: 2.50, 10.53)]. No statistically significant differences were observed among the other four physical activity types in their pairwise comparisons.

**Table 2:** League table on interventions.

Resistance exercise	Aerobic exercise	Mind–body exercise	Multimodal exercise	Stretching exercises	No intervention
Resistance exercise	−4.32 (−9.34, 0.70)	−4.88 (−9.99, 0.23)	−4.92 (−9.77, −0.07)	−5.13 (−9.65, −0.62)	−6.51 (−10.53, −2.50)
4.32 (−0.70, 9.34)	Aerobic exercise	0.56 (−4.03, 5.15)	0.60 (−3.70, 4.90)	0.81 (−3.98, 5.61)	2.19 (−0.83, 5.22)
4.88 (−0.23, 9.99)	−0.56 (−5.15, 4.03)	Mind–body exercise	0.04 (−4.45, 4.53)	0.26 (−4.74, 4.23)	1.64 (−1.83, 5.10)
4.92 (0.07, 9.77)	−0.60 (−4.90, 3.70)	−0.04 (−4.53, 4.45)	Multimodal exercise	0.21 (−3.96, 4.39)	1.60 (−1.47, 4.66)
**5.13 (0.62, 9.65)**	−0.81 (−5.61, 3.98)	−0.26 (−4.23, 4.74)	−0.21 (−4.39, 3.96)	Stretching exercises	1.38 (−2.33, 5.09)
**6.51 (2.50, 10.53)**	−2.19 (−5.22, 0.83)	−1.64 (−5.10, 1.83)	−1.60 (−4.66, 1.47)	−1.38 (−5.09, 2.33)	No intervention

Interventions are labeled on the diagonal. The colored cells distinguish the two triangular directions of comparison: values below the diagonal represent the row intervention minus the column intervention, whereas values above the diagonal show the reverse comparison. Bold values indicate statistically significant effects (95% CI excluding 0). Positive values favor the row-diagonal intervention.

**Figure 6:**
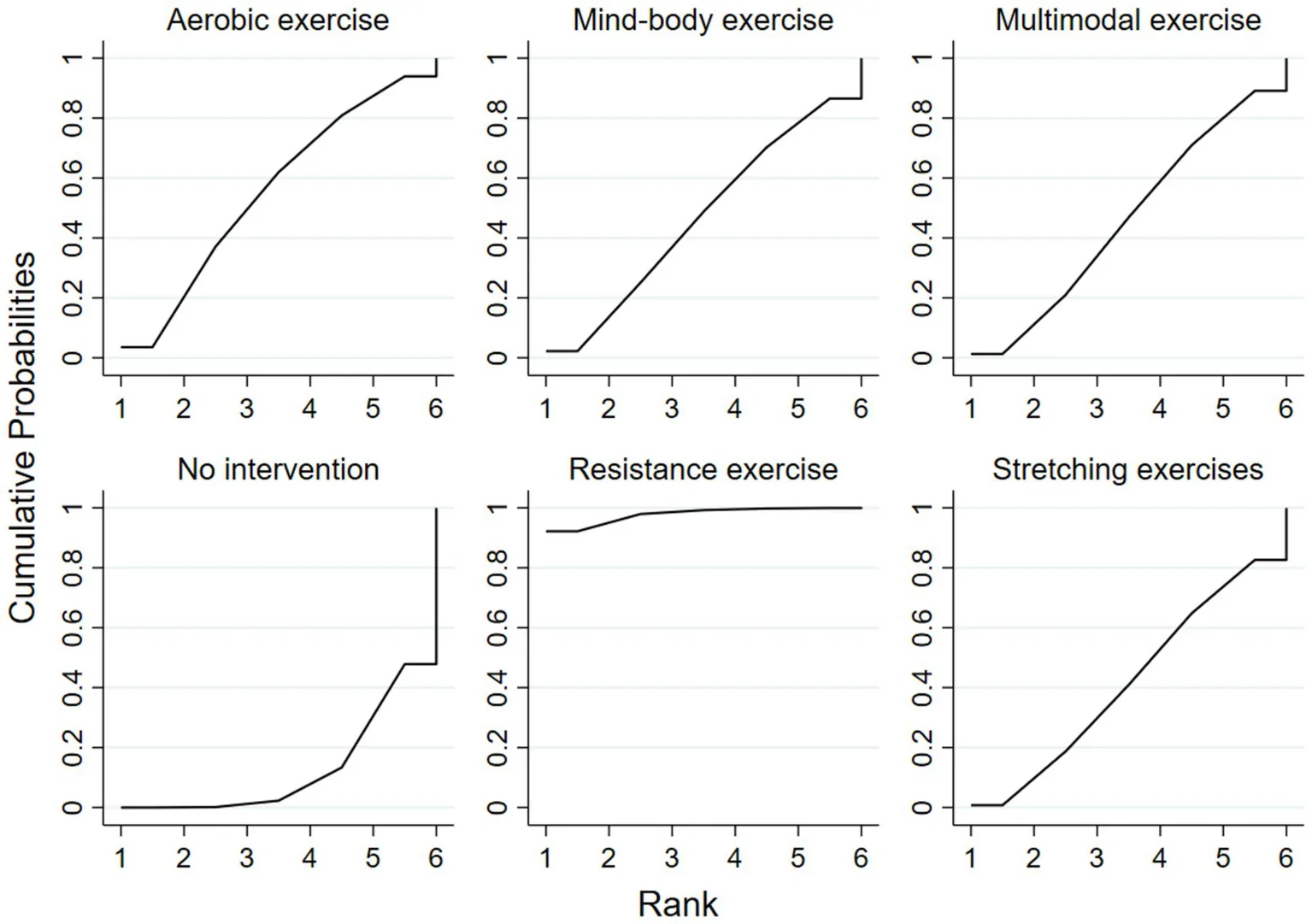
SUCRA plot.

The SUCRA was used to quantitatively rank the relative efficacy of all interventions, with SUCRA values ranging from 0% (least effective) to 100% (most effective) and smaller MeanRank values indicating superior intervention effects. No intervention was included solely as a reference control and not classified as an active intervention. As shown in Table 3 and Figure 6, resistance exercise ranked first with a SUCRA value of 97.8% and a MeanRank of 1.1, demonstrating the greatest efficacy in improving self-esteem. Aerobic exercise ranked second (SUCRA = 55.5%, MeanRank = 3.2), followed by mind–body exercise (SUCRA = 46.6%, MeanRank = 3.7), multimodal exercise (SUCRA = 45.8%, MeanRank = 3.7), and stretching exercises (SUCRA = 41.6%, MeanRank = 3.9). Notably, pairwise comparisons (Table 3) revealed that resistance exercise was significantly superior to multimodal exercise, stretching exercises, and no intervention (all 95% CIs excluded 0), while the differences in efficacy among aerobic exercise, mind–body exercise, multimodal exercise, and stretching exercises were not statistically significant (all 95% CIs included 0).

**Table 3:** Ranking of SUCRA probabilities.

Treatment	SUCRA	MeanRank
Resistance exercise	97.8	1.1
Aerobic exercise	55.5	3.2
Mind–body exercise	46.6	3.7
Multimodal exercise	45.8	3.7
Stretching exercises	41.6	3.9
No intervention	12.7	5.4

Darker orange shading indicates higher SUCRA values and better rankings; lighter orange shading indicates lower SUCRA values and lower rankings. No intervention is the reference control group only and is not an active physical activity intervention.

### 3.5 Publication bias

**Figure 7:**
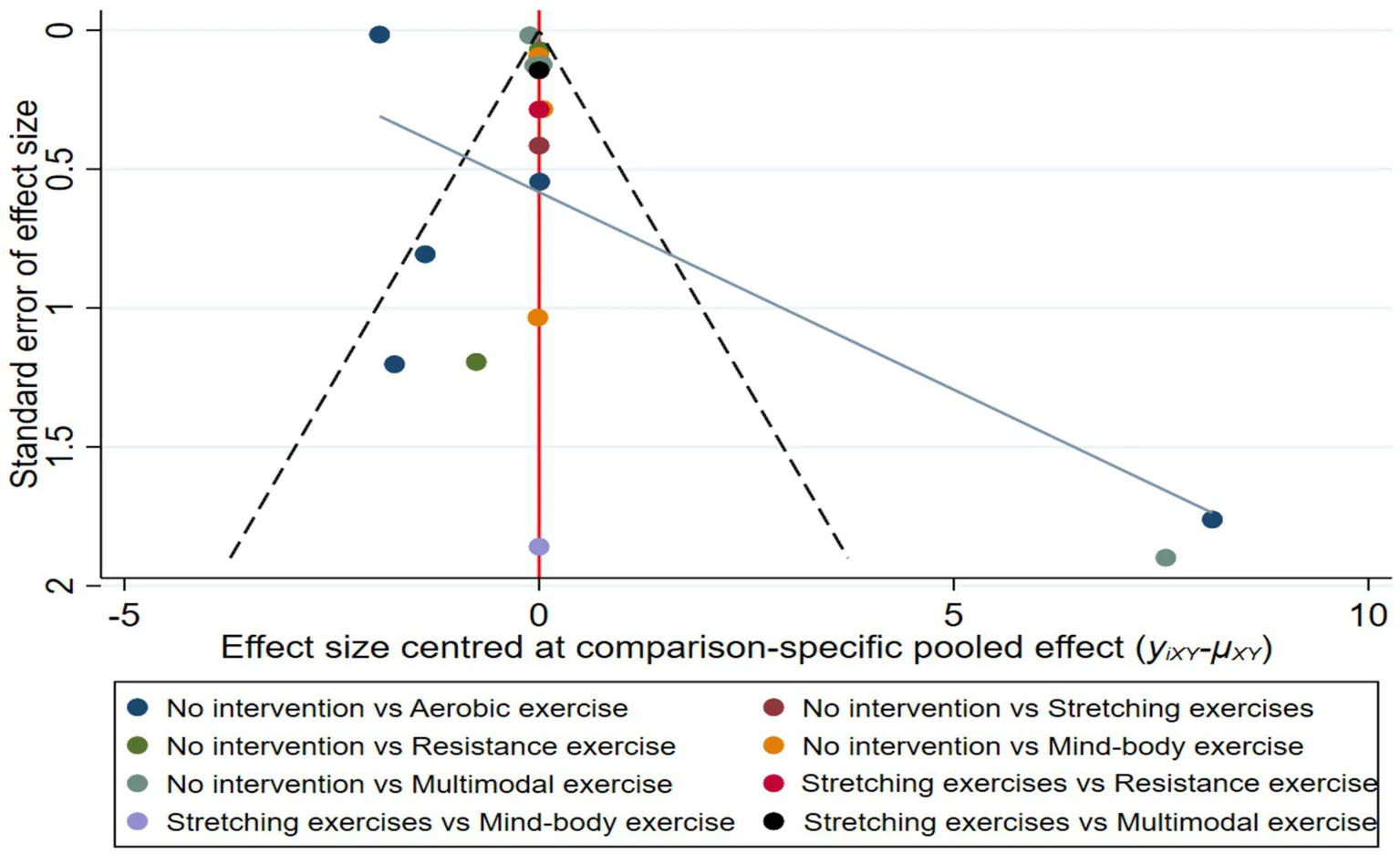
Funnel plot on publication bias.

As shown in Figure 7, publication bias was assessed using a funnel plot. The results indicated that the distribution of studies was generally symmetrical, with no evident publication bias observed. Even if a slight publication bias existed in the original data, its impact was not significant, and the estimated effect sizes remained statistically meaningful, suggesting that the findings are robust and reliable.

## 4 Discussion

This study employed a systematic review and network meta-analysis to comprehensively examine the effects of different types of physical activity interventions on self-esteem in typically developing children and adolescents. Integrating data from 17 RCTs, the results demonstrated that resistance exercise, aerobic exercise, mind–body exercise, multimodal exercise, and stretching all exerted positive effects on self-esteem in this population. Aerobic exercise, mind–body exercise, multimodal exercise, and stretching followed in descending order, each showing significantly greater improvements compared to no intervention. These findings address a critical evidence gap regarding direct comparisons among different exercise modalities and provide high-quality, evidence-based guidance for schools, families, and communities in designing targeted and practical mental health promotion programs for children and adolescents.

Childhood and adolescence are critical periods for the development of self-esteem. Low self-esteem not only exacerbates internalizing issues such as anxiety and depression but also impairs social adaptation and academic performance (45, 46). Compared to pharmacological treatments and professional psychological interventions, physical activity offers advantages such as low cost, ease of promotion, high safety, and broad adaptability. It can naturally be integrated into physical education curricula, extracurricular activities, and daily life, making it more suitable for large-scale implementation in typically developing children and adolescents (47, 48). This study confirms that different types of physical activity effectively improve self-esteem, further strengthening the scientific basis for the concept of “promoting mental health through physical activity” (49). It also provides a feasible path for addressing the current challenges of limited psychological intervention resources and coverage for children and adolescents. Instead, the present findings quantitatively rank the relative efficacy of five exercise types, offering scientific support for the precise selection of intervention strategies.

Resistance exercise had the highest likelihood of boosting self-esteem (SUCRA = 97.8%, MeanRank = 1.1), which is consistent with sports psychology’s self-efficacy theory (29, 41, 42). Its potential effects operate via multiple pathways. Physiologically, it improves muscle strength, bone density and body posture, elevates anabolic hormone levels, and optimizes neural activity to enhance body satisfaction and positive self-perception (50–52). Psychologically, tangible progress in strength and skills builds competence and a strong sense of self-worth (53, 54). These physical and psychological benefits may form a positive cycle. Since we did not verify these mediating mechanisms, further RCTs with repeated measurements are required to confirm the hypotheses. Despite its advantages, resistance exercise is recommended as a core intervention and can be combined with other exercise types to accommodate different preferences and sustain participation.

Aerobic exercise, which follows closely behind, also demonstrates a significant effect on enhancing self-esteem (26–30). Based on existing external theoretical evidence, we speculate that its effects are mainly achieved through emotional regulation pathways: At the physiological level, aerobic exercise improves cardiorespiratory function through sustained rhythmic activity (55), promotes systemic blood circulation and oxygen delivery (56), and effectively upregulates levels of “feel-good” neurotransmitters such as serotonin, dopamine, and endorphins. Simultaneously, it rapidly suppresses excessive secretion of stress hormones (e.g., cortisol), alleviating negative emotions such as anxiety and irritability, thereby creating a stable neuroendocrine environment conducive to self-esteem development (57, 58). At the psychological and social levels, forms of aerobic exercise such as running or jump rope are low-threshold and enjoyable; participation in group activities provides peer recognition and social support, further reinforcing self-identity (59). Compared with resistance exercise, aerobic exercise primarily targets emotional regulation, producing a more moderate but sustained improvement in self-esteem (60). However, it lacks the direct sense of competence afforded by clear strength gains, resulting in a smaller impact on body self-concept and core self-efficacy, which largely accounts for its slightly weaker overall effect (61).

Compared to the previous two forms of exercise primarily based on physiological activation, mind–body exercises exert their effects through a more gentle mind–body regulation pathway, offering unique value in promoting self-esteem (36, 39). At the physiological level, practices such as yoga and Tai Chi combine slow, controlled movements with deep breathing to activate the parasympathetic nervous system, thereby stabilizing and reducing the overactivation of the HPA axis, improving heart rate variability and sleep quality, and alleviating nervous tension and physical fatigue (62, 63). These practices also enhance proprioception and body awareness, reducing negative self-evaluations resulting from physical discomfort (64). At the psychological level, mind–body exercises emphasize the unity of intention and movement, helping children and adolescents cultivate a calm, accepting attitude toward themselves. This is particularly beneficial for emotionally sensitive and easily stressed individuals, as it indirectly consolidates self-esteem by stabilizing physiological states (65, 66). However, due to their relatively low level of physiological activation, mind–body exercises are less likely to result in significant improvements in physical fitness and strength, and their direct impact on self-efficacy is limited. As a result, their overall effect is weaker than that of resistance and aerobic exercises (67).

Multimodal exercise, which integrates multiple movement forms, has long been recognized for its comprehensive physical benefits and higher adherence rates among children, as its diverse and engaging nature effectively reduces exercise monotony (32, 34, 68). Our study confirmed that multimodal exercise does significantly enhance self-esteem in typically developing children and adolescents compared to no intervention, though it ranked fourth among the five active intervention types (34). Critically, our network meta-analysis revealed a key previously unquantified finding: multimodal exercise was significantly less effective than resistance exercise in improving core self-worth (68). This discrepancy likely stems from dispersed training objectives: unlike resistance exercise, which delivers clear, incremental feedback on strength gains that directly reinforce physical self-concept and self-efficacy, multimodal programs spread training efforts across multiple domains, resulting in less pronounced and less immediate feelings of competence (69). These findings clarify the optimal positioning of multimodal exercise: it is best suited as a foundational or complementary intervention, particularly for younger children who thrive on play-based, varied activities, and can be paired with targeted resistance exercise components to maximize self-esteem promotion outcomes.

Stretching exercises, while having a mild effect on self-esteem, offer unique value as a foundational or adjunctive method (40, 41). They alleviate muscle tension, improve circulation, and reduce discomfort from prolonged sitting (70, 71). The relaxed, low-intensity experience can reduce exercise-related anxiety (72). Stretching alone is unlikely to serve as a core strategy but can be integrated into warm-up or cool-down phases of other interventions, or used as a low-threshold option for sedentary or highly anxious youth.

Compared with previous studies that primarily focused on a single type of exercise or generalized effects of physical activity, the present study offers three notable advantages. First, it targets typically developing children and adolescents while excluding confounding factors such as overweight/obesity, developmental disorders, or psychiatric conditions, thereby enhancing the generalizability and specificity of the findings. Second, it employs a network meta-analysis to achieve both direct and indirect comparisons among five categories of exercise, allowing for a quantitative ranking of effect sizes and providing clear guidance for practical implementation. Third, it incorporates large-sample, high-quality RCTs, with robust assessments of publication bias, ensuring the reliability of the conclusions. Overall, this study systematically elucidates the differential mechanisms and hierarchical effects of various types of physical activity on self-esteem in children and adolescents, establishing a critical evidence base for constructing a scientific and precise framework for promoting exercise-based mental health. Moreover, it offers novel insights for the optimization and advancement of public health strategies aimed at supporting psychological well-being in youth.

### 4.1 Strengths and limitations

This study strictly focuses on typically developing children and adolescents, excluding confounding factors like overweight/obesity, developmental disorders and mental health conditions to improve the generalizability and specificity of findings. It adopts an advanced systematic review and network meta-analysis design, enabling direct and indirect comparisons of five physical activity types and quantitative efficacy ranking, filling the evidence gap on the differential effects of exercises on self-esteem. All included studies are high-quality RCTs with complete standardized data. Publication bias tests confirm symmetric distribution and robust conclusions. It also elucidates the physiological, psychological and social mechanisms of exercise-induced self-esteem enhancement, providing a solid theoretical basis for practical applications. However, the study has limitations, particularly methodological ones: First, only three resistance exercise studies were included, and the small sample size may compromise the stability of effect size estimates, especially for direct head-to-head comparisons with other modalities. Second, only English peer-reviewed full-text articles were included, excluding gray literature and non-English publications, which may introduce language bias. Third, the star-shaped evidence network relies heavily on indirect comparisons via the no-intervention control, as most pairwise comparisons lack direct RCT evidence. Fourth, only a frequentist framework was used for network meta-analysis without Bayesian verification, and no sensitivity or subgroup analysis was conducted to test result robustness and explore heterogeneity sources. Fifth, all studies used only no intervention/usual care as controls, lacking comparisons with mainstream non-pharmacological interventions such as cognitive behavioral therapy. Additionally, there were unavoidable heterogeneities in intervention protocols and over a dozen self-esteem assessment tools were used, which may bias effect size aggregation. Most studies only reported short-term effects without long-term follow-up, and were conducted in developed countries, limiting the extrapolation of findings to non-Western and developing regions.

### 4.2 Practical implications

Schools are advised to revise physical education curricula and adopt bodyweight resistance training as a core intervention, combined with aerobic and multimodal exercises. Equipment-free, easy-to-implement resistance programs should also be promoted in families and communities, and physical activity can be integrated into youth mental health public health services as a scalable non-pharmacological intervention. For children with low self-esteem and emotional vulnerability, gentle mind–body exercises and stretching are recommended. Intervention plans should be tailored by age, fitness level, personality and mental state: playful multimodal activities suit younger children, while adolescents can gradually take part in more intensive resistance and aerobic training. Nevertheless, this finding requires cautious interpretation. The superior ranking of resistance exercise is derived from network meta-analysis probabilities and supported by merely three small-sample RCTs. Its advantage over aerobic exercise was not statistically significant (SMD = 4.32, 95% CI: −0.70, 9.34). Further large-scale, head-to-head RCTs are needed to verify this preliminary result and solidify relevant evidence for practical application.

## 5 Conclusion

The results of this systematic review and network meta-analysis confirm that five types of physical activities—resistance exercise, aerobic exercise, mind–body exercise, multimodal exercise, and stretching exercises—effectively enhance self-esteem in typically developing children and adolescents, with outcomes significantly superior to no intervention. Among these, resistance exercise showed the highest probability of being the most favorable intervention (SUCRA = 97.8%), followed by aerobic exercise, mind–body exercise, multimodal exercise, and stretching exercise, respectively. This study provides preliminary evidence for selecting optimal exercise programs. It is recommended that schools, families, and communities consider resistance exercise as a preferred intervention based on current findings, supplemented by other types of physical activities according to context and individual characteristics, to construct a sustainable and tailored exercise intervention system for promoting mental health among children and adolescents. Importantly, the superiority of resistance exercise should be interpreted with caution due to the limited evidence base, and future large-scale head-to-head RCTs are needed to confirm this result.

## Data Availability

The datasets presented in this study can be found in online repositories. The names of the repository/repositories and accession number(s) can be found in the article/supplementary material.
